# Downregulation of transforming growth factor-β type II receptor prohibit epithelial-to-mesenchymal transition in lens epithelium

**Published:** 2012-05-11

**Authors:** Danying Zheng, Tingting Song, Xueying Zhongliu, Mingxing Wu, Jingli Liang, Yizhi Liu

**Affiliations:** State Key Laboratory of Ophthalmology, Zhongshan Ophthalmic Center, Sun Yat-sen University, Guangzhou, China

## Abstract

**Purpose:**

Transforming growth factor-β (TGF-β) is considered to be essential to induce epithelial-to-mesenchymal transition (EMT) which plays central roles in wound healing in ocular fibrotic complication. The present study investigates whether small interference RNAs (siRNAs) targeting the type II receptor of TGF-β (*TβRII*) could be used to minimize the TGF-β action.

**Methods:**

TGF-β receptor type II (*TβRII*) specific siRNAs designed from the Nakamura human gene sequence were used to transfect the cultured lens epithelial cells (LECs). The optimal transfection of scramble siRNA-Cy3 labeled duplexes in cultured LECs were examined by laser scanning confocal microscope and flow cytometry. TβRII protein expression and transcript levels were analyzed by immunofluorescence, western blotting, and real time PCR, respectively. Western blotting was performed to examine protein expression of fibronectin and alpha-smooth muscle actin (α-SMA). Scratch assay was used to determine cell migration. Cell morphology was observed after transfection by inverted microscope.

**Results:**

The optimal transfection rate of scramble siRNA-Cy3 labeled duplexes was efficient in that nearly to 50% in cultured LECs. TβRII specific siRNAs significantly reduced the receptor transcript and protein expression in cultured LECs. The gene knockdown inhibited LECs transdifferentiation, as it abrogated the expression of fibronectin and α-SMA, and retarded cell migration on the scratch assay. In addition, after transfection with *TβRII* specific siRNA, the cultured LECs did not show fibroblast-like shape which was one of the feature signs of EMT. Wound scratch assays indicated that the number of cultured LECs migrated into the wounded area was significantly lower in *TβRII* specific siRNA treated group (12.8±3.27/7.85 mm^2^), compared with normal (57.8±3.06/7.85 mm^2^) and scrambled RNA transfected group (50.8±3.64/7.85 mm^2^; p<0.0001).

**Conclusions:**

Our results provided additional evidence to support that TGF-β pathway was involved in the development of EMT of human posterior capsule opacification, while how TβRII was involved should be further investigated.

## Introduction

As an important refractive media, the ocular lens is transparent and provides part of the refractive power needed to focus images on the retina. Recently, there has been great interest in human posterior capsule opacification (PCO) which are secondary cataracts due to residual lens epithelial cells (LECs) transdifferentiation after cataract surgery [1]. To investigate the mechanisms involved in transformation of LECs may help to prevent and treat this type of cataract.

Epithelial-mesenchymal transition (EMT) is a process in which epithelial cells lose their differentiated phenotypes and change its morphology and transcriptional program to the characteristic of a mesenchymal cell [2]. The results of both cell study and human sample pathological study supported that EMT was an important LECs transformation during wound healing after cataract surgery [3-5].

Transforming growth factor-β (TGF-β) is member of a large metazoan family of multifunctional cytokines involving in a wide range of cellular processes, including cell growth, differentiation, inflammation, and wound healing [6-8]. TGF-β orchestrates the EMT of various epithelia tissues in response to injury [9-11]. Although five members of the TGF-β family have been identified, only TGF-β isoforms 1, 2, and 3 have been detected in mammals with TGF-β2 predominant [12,13].

TGF-β2 was a strong inducer of transformation and pathologic fibrosis of epithelial cells by modulating components of the extracelluar matrix (ECM) [14,15]. This process involves the activation of several EMT-associated target genes and accumulation of fibroblastic/myofibroblastic makers: α-smooth muscle actin (α-SMA) and fibronectin [16-19]. TGF-β signaling is initiated by TGF-β binding to type II receptor of TGF-β (TβRII), followed by its recruitment and phosphorylation of type I receptor of TGF-β (TβRI) [6].

Regulating the biologic activities of residual LECs during the wound healing process may be a potential anti-EMT strategy for cataract surgery, and blocking TGF-β signaling seems promising [20,21]. The use of anti-TGF-β antibody and antisense oligonucleotides to block the TGF-β action for glaucoma surgery has been reported [22-24].

RNA interference (RNAi) can easily and effectively inhibit the expression of a specific gene by which double stranded RNA triggers the destruction of mRNAs sharing the same sequence [25]. Therefore, RNAi can prevent synthesis of a protein encoded by the target mRNA [26]. Recently, RNAi-mediated gene silencing has been shown to be efficient in mammalian cells, and this has led to the increasing feasibility of RNAi technology for the therapy of certain human diseases [27].

Therefore, this study aimed to investigate whether the RNAi strategy targeting *TβRII* could effectively manipulate the activation and proliferation of LECs during the EMT process after cataract surgery.

## Methods

### Cell culture

SRA01/04 cells (a human lens epithelial cell line from Shang Fu professor, ATCC, Manassas, VA) were cultured in Dulbecco's Modified Eagle Medium (DMEM; GIBCO, Grand Island, NY) supplemented with 10% fetal bovine serum (FBS; HyClone, Logan, UT), 2.2 g/l sodium bicarbonate, and 10 mmol/l HEPES 2-[4-(2-Hydroxyethyl)-1-piperazinyl]ethanesulfonic acid) buffer in a humidified 37 °C incubator with 5% CO_2_ atmosphere.

### TβRII siRNA sequences design

Three pairs of siRNAs targeting *TβRII* were designed according to the *TβRII* sequence in GenBank (NM_003242). All the duplex sequences and target sequences of these siRNAs identified were BLASTed against the GenBank database. Three siRNA sequences that cover different regions of the *TβRII* coding sequence without homology to the non-*TβRII* sequence were custom synthesized by Ribobio (Guangzhou, China). The target sequences (5′ to 3′) and the siRNA duplexes for these three ones, designed as hT1, hT2, and hT3, are shown in Table 1. In addition, a nonspecific, scrambled siRNA duplex with Cy3 labeled, served as a control to determine optimal conditions for siRNA transfection, were also synthesized by Ribobio.

**Table 1:** Target and duplex sequences for *TβRII* specific siRNAs.

**siRNA duplex**	***TβRII* sequence (5′-3′)**	**siRNA duplex sequences**
hT1	TCCTGCATGAGCAACTGCA	(5′-3′) 5′ UCCUGCAUGAGCAACUGCA dTdT 3′
		(3′-5′) 3′ dTdT AGGACGUACUCGUUGACGU 5′
hT2	GCATGAGAACATACTCCAG	(5′-3′) 5′ GCAUGAGAACAUACUCCAG dTdT 3′
		(3′-5′) 3′ dTdT CGUACUCUUGUAUGAGGUC 5′
hT3	GGCCAAGCTGAAGCAGAAC	(5′-3′) 5′ GGCCAAGCUGAAGCAGAAC dTdT 3′
		(3′-5′) 3′ dTdT CCGGUUCGACUUCGUCUUG 5′

### Transfection of scramble siRNA-Cy3 labeled duplexes to optimize transfection conditions

Cells were plated in 24-well culture plate at 1×10^4^ cells/well to 30% confluence overnight. For transfection, the culture medium was removed from the cells and was replaced with 500 µl Opti-MEM® I medium (GIBCO, invitrogen, Guangzhou, China) for each well. Lyophilized siRNAs were dissolved in Rnase-free H_2_O (pH=7.4) and stored at −20 °C before use. Lipofectamine™ 2000 reagent (Invitrogen) were kept for 5 min at room temperature before mixing with Cy3-conjugated scrambled siRNA. The cultured LECs were transfected with scrambled (control) siRNA (20, 40, 50, 60 or 80 nM final concentration) using Lipofectamine™ 2000 reagent, following the manufacturer's protocol. The cells were transfected for 6 h for analyses. Also as controls, cells were either untreated or treated with Lipofectamine™ 2000 reagent.

### Transfection of Target siRNA duplexes

The cultured LECs were transfected with *TβRII* specific siRNA duplex (80 nM final concentration) or scrambled siRNA (80 nM) as above. The cells were harvested 24, 48, or 72 h after transfection for analyses. Also as controls, cells were either untreated or treated only with Lipofectamine™ 2000 reagent.

### Analysis of transfection optimization

Transfected with scrambled siRNA (20, 40, 50, 60 or 80 nM final concentration) after 6 h, cells were rinsing three times with PBS and replaced with complete culture medium. Images were photographed with Zeiss LSM 510 confocal laser scanning device (Zeiss, Oberkochen, Germany). The group of cells transfected with scrambled siRNA (60, 80 or 100 nM) were trypsinized, harvested and resuspended in PBS at a density of 1×10^6^ cells/ml for flow cytometry (BD Bioscience, San Jose, CA) analysis with a 550-nm laser. Treatments were performed in triplicate and the results are displayed as the average transfected percentage of total cell number.

### Detection of *TβRII* mRNA expression by real time PCR

Total RNA was extracted using Trizol ® Reagent (Invitrogen, Carlsbad, CA) and reverse transcribed for 45 min at 45 °C using TaKaRa RNA PCR Kit (AMV) Ver.3.0 (TaKaRa, Dalian, China). Real-time PCR reactions was performed in an ABI 7500 real time PCR system (Applied Biosystems, Invitrogen, Guangzhou, China) using the following PCR parameters: 93 °C for 3 min followed by 30 cycles at 93 °C for 30 s, 55 °C for 30 s, and 72 °C for 45 s. Results were evaluated on the basis of mRNA copy number/1 μg of total RNA and normalized by β-actin (*ACTB*) as an internal control. Each sample was tested at least three times. The sequences of the primers were as follows: *TβRII*, 5′-ACC ACC AGG GCA TCC AGA T-3′(sense), 5′-TGA AGC GTT CTG CCA CAC A-3′ (antisense); *ACTB*, 5′-GCA TGG GTC AGA AGG ATT CCT-3′ (sense), 5′-TCG TCC CAG TTG GTG ACG AT-3′ (antisense).

### Immunofluorescence staining

After transfection, cells were fixed in 4% paraformaldehyde solution, permeabilized with 0.1% Triton X-100, and stained with rabbit anti-TβRII antibody (1:50, TGF-β RII (C-16):sc220; Santa Cruz Biotechnology Inc., Santa Cruz, CA) and FITC conjugated goat anti-rabbit IgG (1:32; Southern Biotechnology, Birmingham, AL) . The nuclei of the cells were counterstained with Hoechst 33342 (5 µg/ml; Sigma, Guangzhou weiga science and technology company, Guangdong, China). Images were captured with a Zeiss LSM 510 confocal laser scanning device.

### Western blotting

After siRNA transfection, HLECs were lysed in an ice-cold Triton lysis buffer (50 mM, pH 7.5; Invitrogen). Equal amount of protein (20 μg/lane) was resolved on a 12% sodium dodecyl sulfate (SDS)-polyacrylamide gel. The proteins were then transferred to polyvinylidene fluoride membranes (PVDF membranes; Millipore, Guangzhou weiga science and technology company) for probing with rabbit anti-TβRII antibody (1:1,000) and horseradish peroxidase (HRP) conjugated goat anti-rabbit IgG (1:5,000; Santa Cruz Biotechnology).

In addition, cells (48 h after transfection) after normalizing against the protein content in cell lysates, equal amount of protein (20 µg/lane) was resolved in 12%SDS–PAGE. The proteins were then transferred to PVDF membranes for probing with rabbit anti-human fibronectin monoclonal antibody (1:1,000; Epitomics, Guangzhou weiga science and technology company), rabbit anti-human alpha-smooth muscle actin (α-SMA) polyclonal antibody (1:300, No: ab5694; Abcam, Guangzhou weiga science and technology company) and HRP (1:5,000), respectively. GAPDH (glceraldehyde 3-phosphate dehydrogenase) staining served as an internal control.

Signals were detected by chemiluminescence and analyzed by densitometry using Image J software (image processing and analysis in Java).

### Cell migration assay

Cultured LECs after transfection were scratched with a sterile P20 pipette tip. After cultures were at approximately 90% confluence, a standard 200-μl yellow tip was drawn across the center of each well to produce the wound. The migration of cells into the wound was examined by inverted microscope at another 48 h after transfection. At least 10 microscopic fields were analyzed and the average cell number that migrated into the wound area was calculated.

### Cell morphology

SRA01/04 cells at 48 h after transfection were observed. The morphology of each group was examined by inverted microscope.

### Statistical analysis

Statistical analysis was performed with a statistical software package (SPSS for Windows, version 16.0, SPSS, Chicago, IL). The data of each group were compared and are expressed as the mean±SD, with p<0.05 taken to indicate significance. Statistical comparions between the groups were performed using analysis of variance (AVOVA) with the Bonferonni-Dunn adjustment.

## Results

### Optimize conditions for scramble siRNA-Cy3 labeled duplexes transfection

**Figure 1:**
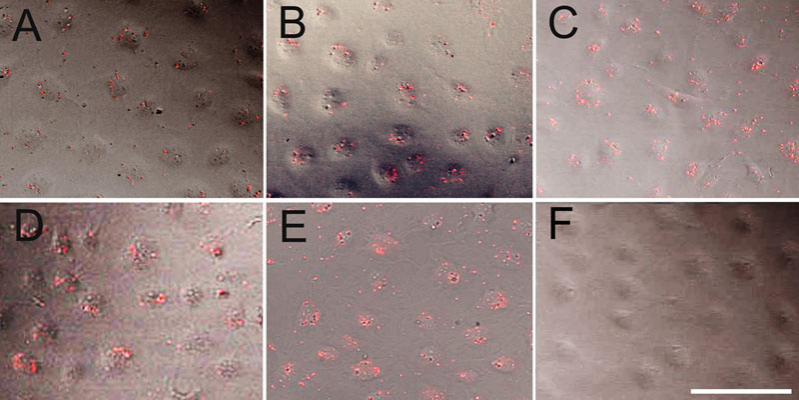
Qualitative analysis of optimal transfection of different concentration of Cy3-labeled scrambled siRNA for SRA01/04 cells. Distribution of Cy3-labeled scrambled siRNA (red fluorescence) in SRA01/04 cells at 6 h after scrambled siRNA transfection with a final concentration gradient of 20 (**A**), 40 (**B**), 50 (**C**), 60 (**D**), or 80 nM (**E**). Cells incubated with Lipofectamine™ 2000 reagent alone (no siRNA) served as blank control which did not show fluorescence appearance (**F**). Bar: 25 μm.

**Figure 2:**
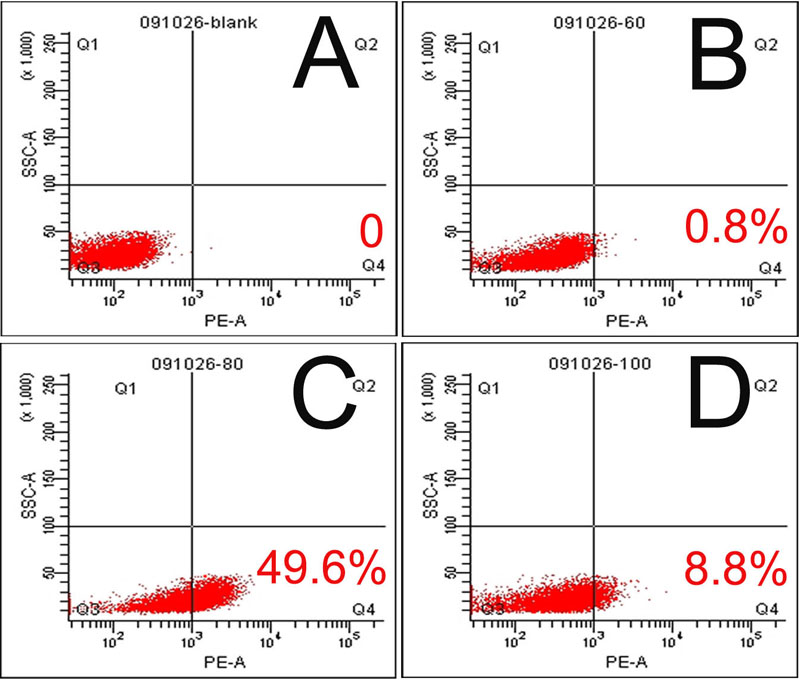
Quantitative analysis of optimal transfection of different concentration of Cy3-labeled scrambled siRNA for SRA01/04 cells. The Cy3-labeled scrambled siRNA transfection rates of SRA01/04 cells after incubating for 6 h were evaluated by flow cytometry. **A**: Blank group: 0%. **B**: 60 nM Cy3-labeled scrambled siRNA: 0.8%. **C**: 80 nM Cy3-labeled scrambled siRNA: 49.6%. **D**: 100 nM Cy3-labeled scrambled siRNA: 8.8%.

The optimal ratio of scrambled siRNA transfection was analyzed by laser scanning confocal microscopy with Cy3 staining (Figure 1). Red punctuate staining of scrambled siRNA on the surface of transfected cells were increased gradually in number or intensity (Figure 1A-E). Transfection efficiency was also evaluated by measuring the percentage of cells containing Cy3-conjugated siRNA using flow cytometry analysis (Figure 2). The optimal transfection rate was efficient in that nearly to 50% with 80 nM final concentration (Figure 2C). Both methods revealed that the effect of cells displayed red fluorescence of the luciferase siRNA was in a dose dependent manner. The effect of varied concentration of scrambled siRNAs were tested. Scrambled siRNA with 100 nM induced elongation, apoptosis and decreased viability of cells (data not shown).

### Suppression of TβRII protein and mRNA expression

**Figure 3:**
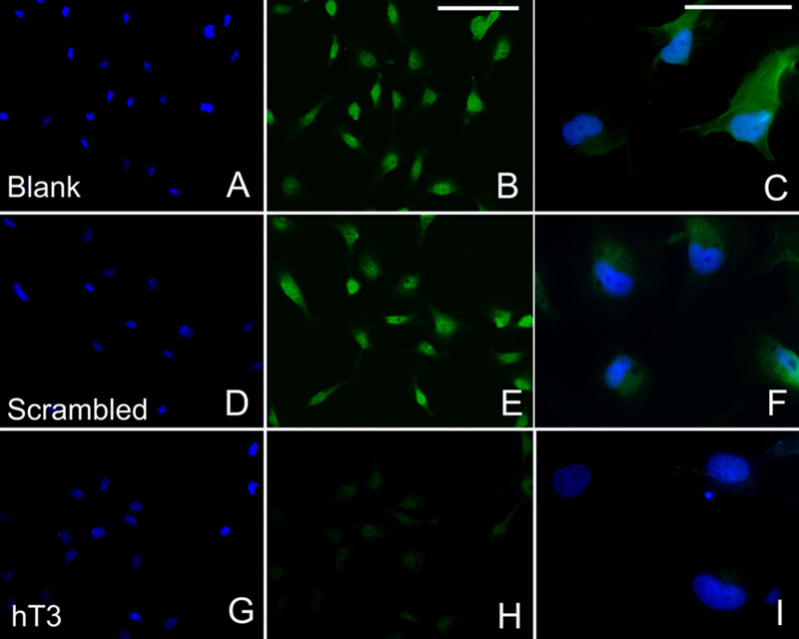
Transfection with targeting siRNAs suppressed TβRII protein expression by immunofluorescence staining. Untreated SRA01/04 cells (**A**-**C**), SRA01/04 cells treated with 80 nM scrambled siRNA for 48 h (**D**-**F**), or treated with 80 nM hT3 (**G**-**I**) were observed under immunofluorescence microscope. Staining of nuclei with Hoechst 33342 (blue) is shown in **A**, **D**, and **G**. TβRII expression is shown in **B**, **E**, and **H**. Compared with the control cells (**B** and **E**), TβRII staining of cells treated with hT3 siRNA was weaker (**H**). The morphology after the siRNA treatment were somewhat altered. Note the exclusively cytoplasmatic localization of TβRII expression (**C**, **F**, and **I**). Bar: 25 μm.

Immunofluorescence showed punctuate staining of TβRII on the surface of untreated control HLECs (Figure 3B). The staining was also observed in the cytoplasm, particularly in the perinuclear area. When treated with 80 nM hT3 for 48 h, the TβRII staining intensity was markedly reduced (Figure 3H). At 80 nM, hT1 and hT2 did not markedly suppress the TβRII staining intensity. The inhibitory effect was observed in varied degrees for all three siRNAs with other concentrations (60 and 100 nM) and time points (24 and 72 h). This response however was not evident at the lowest concentration (60 nM) and the shortest time point (24 h) tested. Overall, hT3 showed the greatest inhibition effect among the three sequences. Cells treated with scrambled siRNA (Figure 3E) exhibited a similar staining intensity and pattern as the untreated control cells (Figure 3B).

**Figure 4:**
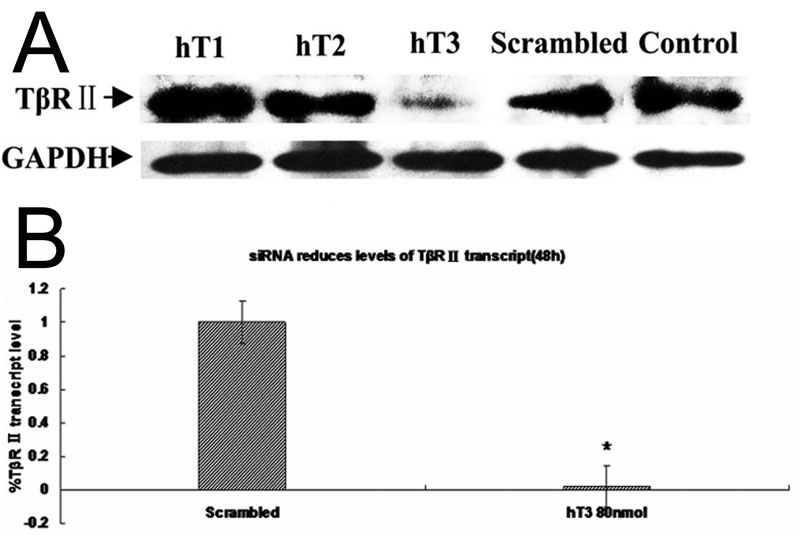
Effect of targeting siRNAs suppressed TβRII protein and mRNA expression by western blot and RT–PCR. **A**: siRNAs suppress TβRII protein expression in SRA01/04 cells. Total cell lysates from SRA01/04 cells treated with hT1, hT2, and hT3 siRNA or control, scrambled siRNA for 48 h were separated on 12% SDS polyacrylamide gels and immunoblotted with a TβRII specific antibody. Lane 5 contains lysate from cells incubated with Lipofectamine™ 2000 reagent alone (no siRNA). Lane 4 contains lysate from cells treated with 80 nM scrambled siRNA. Lanes 1, 2, and 3 contain lysates of cells treated with hT1,hT2, and hT3 siRNA at a final concentration of 80 nM. The TβRII band is indicated. **B**: siRNA reduces levels of *TβRII* transcript. RNA was prepared from cells treated with scrambled siRNA or hT 3 siRNA for 48 h at 80 nM/ concentration. Following conversion to cDNA, the samples were analyzed by real time PCR using primers to *TβRII*. The bar graphs show the mean expression levels of *TβRII* transcript normalized to *ACTB* relative to that of scrambled siRNA treated controls. Asterisks indicate that the data are significantly different from the scrambled controls (p<0.0001, n=3, one-way ANOVA, Dunnett-test).

Western blotting (Figure 4A) yielded a 72 kDa band immunoreactive to anti-TβRII in the vehicle treated control and scrambled siRNA transfected samples. At 48 h, 80 nM hT3 siRNA induced marked decreased signal intensity for TβRII compared with vehicle or scrambled RNA treated controls. Densitometric analysis indicated 80%–85% reduction of TβRII protein expression in hT3 siRNA treated samples. The hT1 and hT2 group exhibited a similar staining intensity and pattern as the untreated control cells.

*TβRII* transcript levels were examined by real time PCR using *ACTB* to normalize the data. Finger 4B showed the mean normalized *TβRII* gene expression as determined on the basis of mRNA copy number/1μg of total RNA. Compared with scrambled siRNA, hT3 at 80 nM significantly reduced the level of *TβRII* transcripts at the 48 h time points (p<0.0001).

### *TβRII* siRNA downregulates myofibroblast markers of α-SMA and fibronectin protein expression

**Figure 5:**
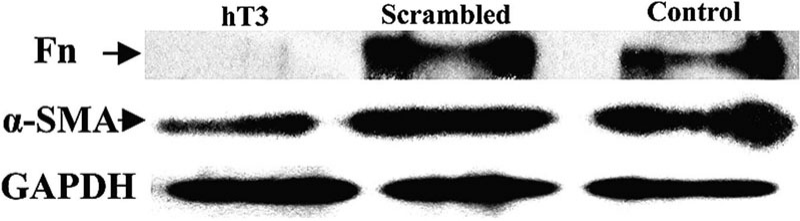
*TβRII* siRNA interferes with fibronectin and alpha-smooth muscle actin (α-SMA) production. The level of fibronectin secreted and α-SMA expression to the culture media was analyzed by western blotting. Samples were from cultures untreated (lane 3), treated with scrambled siRNA (lane 2), or 80 nM (lane 1) hT3. The fibronectin (Fn) and α-SMA bands are labeled.

Western blotting was performed to determine whether siRNAs inhibit the fibronectin and α-SMA protein expression which was a reflection of decreased myofibroblast markers secretion. HLECs were transfected for 48 h. Proteins in the media were subjected to electrophoresis. A 220 kDa fibronectin and a 34 kDa α-SMA (Figure 5) were observed in all samples. Treatment with 80 nM hT3 resulted in decreased fibronectin and α-SMA protein expression (Figure 5). The siRNA elicited a great effect with 80 nM hT3 (89% and 57% reduction of fibronectin and α-SMA protein expression than the scrambled siRNA treated sample).

### *TβRII* siRNA inhibits the migration of HLECs in the wound-healing assay

**Figure 6:**
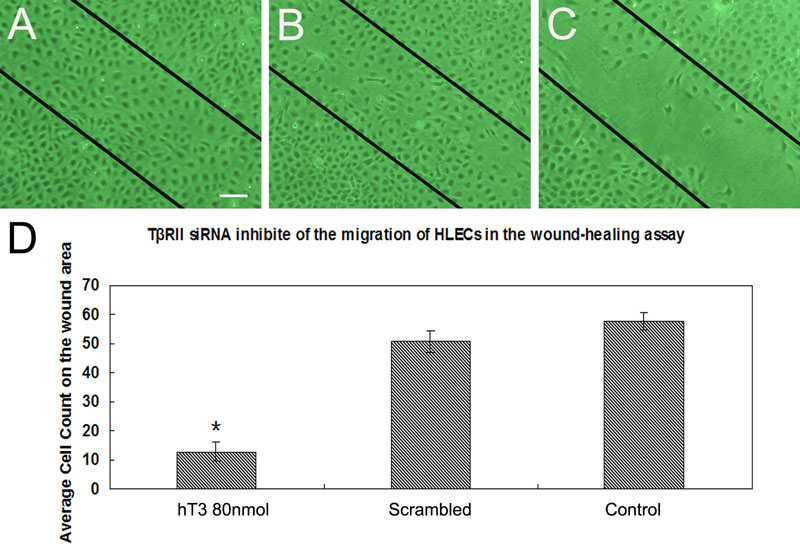
*TβRII* siRNA blocks SRA01/04 cells migration. Inverted microscope images demonstrating migration of HLECs (**A**), cells treated for 48 h with scrambled siRNA (**B**), or cells treated for 48 h with 80 nM hT3 (**C**). Black straight lines mark the wound edges. **D**: Bar graph showing mean cell numbers migrating to wound area in each specimen (n=10); error bars represent the standard error of the mean. Asterisks denote values significantly different from those of scrambled siRNA treated controls (p<0.0001). Experiments were repeated 3 times, yielding similar results. The bar represents 25 μm.

Wound scratch assays indicated that HLECs migrated into the wounded area (Figure 6 A-C). Within 48 h, average of cell numbers that migrated to the wound area for untreated control and scrambled RNA transfected groups were 57.8±3.06/7.85 mm^2^ and 50.8±3.64/7.85 mm^2^ (Figure 6D), respectively. By contrast, 80 nM hT3 (12.8±3.27/7.85 mm^2^; Figure 6D) transfected cells was significantly decreased (p<0.0001).

### *TβRII* siRNA influence cell morphology

**Figure 7:**
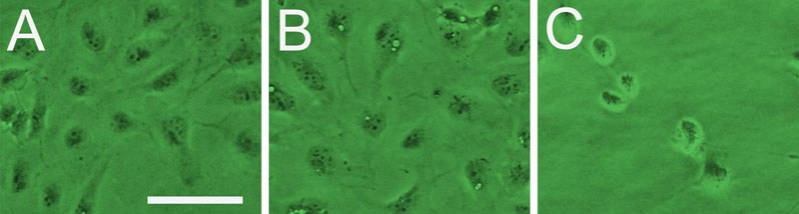
*TβRII* siRNA inhibits the morphological fibroblast-like change of SRA01/04 cells. Inverted microscope images observing cell morphology of HLECs (**A**), cells treated for 48 h with scrambled siRNA (**B**), or cells treated for 48 h with 80 nM hT3 (**C**). Untreated control and scrambled RNA transfected cells were proliferated adhesively (**A** and **B**). By contrast, the proliferation of cells that transfected with 80 nM hT3 cells (**C**) was significantly suppressed. Transfected HLECs showed apoptotic changes such as condense of nucleus and lessened volume relative to untreated control and scrambled RNA transfected cells. The latter exhibited a fibroblast-like morphology obviously. The bar represents 25 μm.

Cells after 48 h transfection was observed by inverted microscopy. Untreated control and scrambled RNA transfected cells were proliferated adhesively (Figure 7A,B). By contrast, the proliferation of cells that transfected with 80 nM hT3 cells (Figure 7C) was significantly suppressed. Transfected HLECs showed apoptotic changes such as condense of nucleus and lessened volume relative to untreated control and scrambled RNA transfected cells. The latter exhibited a fibroblast-like morphology obviously.

## Discussion

The present results showed that the change of fibroblast-like morphology and the migration of human lens epithelial cells were remarkably prohibited after transfection with specific siRNA of *TβRII*, as well as the downregulation of myofibroblast markers of α-SMA and fibronectin protein expression. Currently, the criteria for defining an EMT in vitro have varied in different studies, but it is generally accepted that it involves the acquisition of a mesenchymal morphology and mesenchymal markers. Therefore, the present siRNA of *TβRII* may be regarded to possibly lead to a prohibition of EMT in the PCO.

Cataract surgery can induce a wound-healing response in the lens that leads to PCO which is believed to arise from the proliferation, migration, and EMT of residual lens cells. TGF-β2 has recently proved to be a ubiquitous factor associated with wound-healing response and influence LEC proliferation, migration, and EMT [16,28]. TGF-β2 initiates cellular and molecular changes in lens cells that are relevant with PCO development, including myofibroblast formation, wrinkling of the lens capsule, identification of fibroblast markers, and deposition of ECM [29-31]. However, to our knowledge, there were no previous reports about the relationship between the receptor of TGF-β and the change of lens cells in the research of development of EMT of PCO.

Our results were consistent with previous reports. In the Liu et al. [29] study, TGF-β induced lens epithelial cells in explants to undergo an extensive and rapid elongation with features that distinguished it from fibroblast growth factor (FGF)-induced fiber differentiation, and TGF-β also induced accumulation of extracellular matrix, capsule wrinkling, cell death by apoptosis, and distinctive arrangements of cells. In the Hales et al. [5] study, TGF-β induced rapid cell elongation and formation of characteristic spindle-shaped cells which contained alpha-smooth muscle actin, a marker for myofibroblastic cells and a protein not normally found in the lens. Similarly, numerous in vitro and in vivo studies indicate that this TGF-β-induced EMT is part of a wound healing response in lens epithelial cells and is characterized by induced expression of numerous extracellular matrix proteins (laminin, collagens I, III, tenascin, fibronectin, proteoglycans), intermediate filaments (desmin, alpha-smooth muscle actin) and various integrins (alpha2, alpha5, alpha7) [32].

Ocular fibrotic wound response is a major cause of impaired vision and blindness, and excessive post-operative scarring often leads to failure of cataract surgery. Some previous studies have tried to use antimitotics or antimetabolites to control postoperative PCO [33,34], however, severe blinding complications including hypotony, maculopathy and intraocular infection were observed even after antimitotics use extraocularly in the clinic [35-37]. Additionally, several experimental attempts have been made to find an appropriate therapeutic target to prevent PCO, such as proteasome inhibitors MG-132, lactacystin [38] or gene transfer [39,40], but none has proven effective either because of its toxic effect on other ocular tissues or because of only partial or differential effect on the major causes of PCO, such as LEC proliferation, migration, and transdifferentiation. However, in the recent ten years, RNA interference has become an essential method for in vitro knockdown of target gene and has been used experimentally to prevent ocular neovascularization and inflammation [41-43]. Antisense oligonucleotides or ribozymes for TGF-β were shown to be effective in wound healing by animal and cell studies [24,44,45]. Therefore, as a potential therapeutic way for control of EMT for PCO, RNA interference of TGF-β or TβRII to block the pathway deserved a further investigation.

There were pros and cons in this study. The present study took advantages in the novelty of research of the relationship between inhibition of the receptor for TGF-β, i.e., the TβRII, and the change of lens epithelial cells, and was also prior by using of high efficient RNAi. However, due to the absence of animal study and due to the complexity of EMT, our study failed to give a comprehensive explanation of how TβRII was involved in the development of EMT in PCO. Further studies are required.

In summary, the current study demonstrates that downregulation of the *TβRII* mRNA by application of siRNA inhibit the expression of myofibroblast markers (fibronectin and α-SMA), the morphological fibroblast-like change and the migration of human lens epithelial cells. Our results provided additional evidence to support that TGF-β pathway was involved in the development of EMT of human posterior capsule opacification, while how TβRII was involved should be investigated in the further study.
